# Coarse-Grained Multifractality Analysis Based on Structure Function Measurements to Discriminate Healthy from Distressed Foetuses

**DOI:** 10.1155/2013/152828

**Published:** 2013-12-17

**Authors:** Souad Oudjemia, Amira Zaylaa, Salah Haddab, Jean-Marc Girault

**Affiliations:** ^1^University of Mouloud Mammeri, Tizi-Ouzou, Algeria; ^2^Signal & Imaging Group, University François Rabelais of Tours, UMR INSERM U930, PRES Loire Valley University, 7 Avenue Marcel Dassault, 37200 Tours, Cedex, France

## Abstract

This paper proposes a combined coarse-grained multifractal method to discriminate between distressed and normal foetuses. The coarse-graining operation was performed by means of a coarse-grained procedure and the multifractal operation was based on a structure function. The proposed method was evaluated by one hundred recordings including eighty normal foetuses and twenty distressed foetuses. We found that it was possible to discriminate between distressed and normal foetuses using the Hurst exponent, singularity, and Holder spectra.

## 1. Introduction

Foetal distress is often the result of reduction in respiratory exchange between the mother and the foetus. In most cases, foetal distress is strongly related to intrauterine growth retardation [1]. Early identification of distressed status from heart rate variability is highly important since it can help the obstetrician to decide on immediate delivery by caesarean section.

The value of analysis of heart rate variability (HRV) to provide a means of diagnosis and prognosis of heart disease is now well established. HRV time series has now become the elementary basis from which most analyses and processes are operated.

Due to the nonstationary and nonlinear nature of HRV time series, many recent studies have tried to take full advantage of the nonlinear nature of heart rate variability by analysing the complexity of time series. This complexity analysis of the foetal heart rate (FHR) that has its roots in adult HRV research can be conceived of in many ways. However, it was probably the scale invariance properties observed through power law spectral density [2] that was the triggering element for several studies based on the multiscale analysis of HRV [3, 4]. Among the overall complexity descriptors, entropy descriptors [3, 5, 6] with fractal dimension estimators [7–10] were probably the first “nonconventional” tools used to study FHR. Certain studies even used multifractal features of FHR. The research studies by Ivanov et al. were probably the first to demonstrate multifractality in cardiac dynamics as well as in physiologic dynamics in general [11–13]. These seminal studies were then followed by researches such as [14–17], to name but a few.

The starting point of the present study was based on two approaches, the first being that of Wang et al. [15] focusing on the multifractal analysis of adult ECG signals with a coarse-graining approach initially proposed by [18].

The second approach was based on the studies by [19, 20] and more recently those of [16, 21, 22] that themselves used a method based on a structure function [23] of a time series in order to extract multifractal indicators.

In response to these two kinds of research, we investigated a coarse-grained multifractal analysis of the foetal heart rate in order to discriminate healthy from distressed foetuses.

Although the present study has certain similarities to those proposed by Wang et al. [15], our study was different in two ways. First, unlike the study based on a partition function proposed by [15], our study was based on a structure function. The second difference was that our coarse-graining analysis was performed on the foetal heart rate, whereas that proposed by [15] was evaluated on adult electrocardiograms.

Our study aimed to improve the differentiation between normal and distressed foetuses by investigating the time scale dependency of the multifractal features of the FHR in depth. To do so we investigated the multifractal analysis originating from a structure function from a coarse-graining point of view.

To demonstrate the value of our approach, we tested the proposed method on a dataset derived from normal and distressed foetuses.

## 2. Materials

Our system comprised a personal computer and a Doppler ultrasound unit. The latter device contained three groups of four transducers and a Doppler acquisition board. The transducers exploring the foetal heart were nonfocused and monoelement. The transducers placed on the mother's abdomen were circular in shape, with a diameter of 13.5 mm and an acoustic power of 1 mW/cm^2^. Each transducer transmitted a sinusoidal pulse at 2.25 MHz with a pulse repetition frequency of 1 kHz. The wave was propagated through the mother's abdomen towards the foetal heart.

The backscattered signal was converted into an electrical signal and amplified to compensate for the attenuation of 1 dB/cm/MHz. The signal was then demodulated in phase (I) and quadrature (Q).

The Doppler signals were acquired at CHRU “Bretonneau” Tours, France. The consent of each patient was obtained and the study was approved by the Ethics Committee of the Clinical Investigation Centre for Innovative Technology of Tours (CIC-IT 806 CHRU of Tours). All patients were over eighteen years of age and pregnancies were single. One hundred examinations (eighty normal foetuses and twenty distressed foetuses) were recorded in this study. Gestational ages of foetuses ranged from 25 to 39 weeks were monitored for 30 minutes. FHR was evaluated as proposed by [24, 25], that is, every 250 ms, yielding 7200 samples for a recording of 30 minutes.

## 3. Methods

As previously reported, the foetal heart rate was estimated in real time from ultrasound Doppler signals [24, 25] and then recorded. The coarse-graining from HRV recordings procedure was performed offline. Then segmentation was applied. Scaling factors and multifractal spectra were subsequently evaluated using the structure function (see the scheme in Figure 2).

### 3.1. Coarse-Grained Analysis

Each time series *x*(*n*) composed of *M* = 7200 points was analysed from FHR recordings. Multiscale analysis was introduced to capture the fluctuations present in the time series at different scales. This method consisted of evaluating approximate versions of the original time series from a local average of neighbouring points. This procedure is named “coarse-grained” [18]. The new reduced time series composed of *M*/*α* samples at scale *α* was written as

(1) $$\begin{array}{c} y_{\alpha }\left(k\right)=\frac{1}{\alpha }\sum_{i=(k-1)\alpha +1}^{k\alpha }x_{i}, \end{array}$$

for 1 ≤ *k* ≤ *M*/*α*, *y*_1_(*k*) = *x*(*k*) being the original time series.

**Figure 1:**
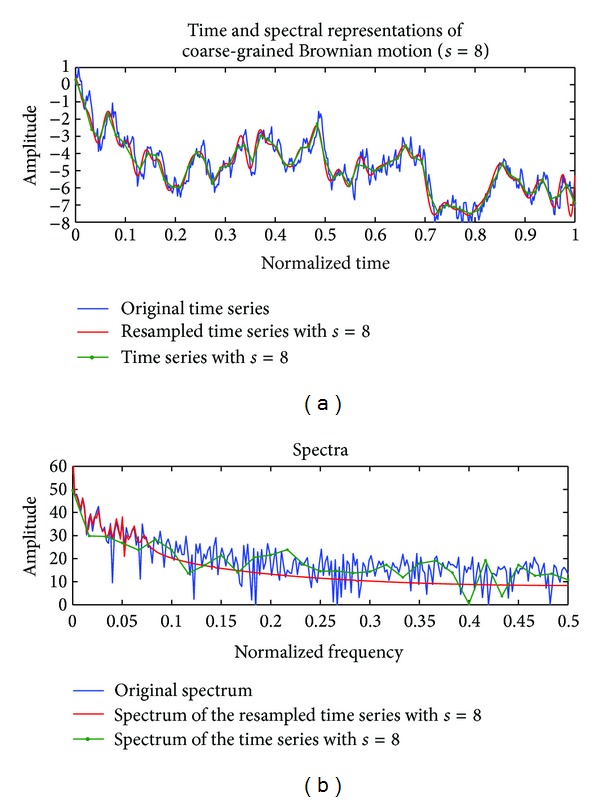
Time and spectral representations of a Brownian motion. (a) Original time series superimposed on the coarse-grained time series (*α* = 8) and the resampled coarse-grained time series (*α* = 8). (b) Spectrum of each time series depicted in (a).

**Figure 2:**
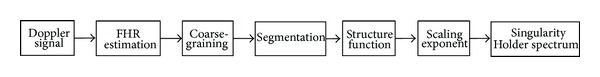
Scheme of different processes used to calculate coarse-grained multifractal descriptors.

Figure 1 sets out the time and the spectral representations of coarse-grained Brownian motion time series used to calculate one of the effects resulting from the coarse-grained procedure. The time and the frequency were normalized.  Figure 1(a) shows the original time series superimposed on the coarse-grained time series with *α* = 8 and the resampled coarse-grained time series with *α* = 8. Note that a resampled coarse-grained time series was an interpolated and filtered time series by a factor *α*. The resampled coarse-grained times series was composed of *M* samples.

Figures 1(a) and 1(b) show clearly that the coarse-grained time series were filtered time series. It can be claimed from these outcomes that the reducing duration of each coarse-grained times series is a side effect that can be avoided by resampling. In the following the multifractal descriptors were evaluated from resampled coarse-grained time series.

As previously shown by [26], the more the fBm was filtered, the more the filtered fBm was regular: it can therefore be claimed that the higher the scale factor *α*, the higher the Hurst exponent *H*. In the study by [27], it was shown that the coarse-graining affected the anticorrelated time series (*H* < 0.5) in a more pronounced way than the correlated time series (*H* > 0.5). By supposing the coarse-grained effect to be a low pass filtering effect, we suggest that this can be understood in the following way.

- For *H* < 0.5, fBm has several high frequency components that can be removed by the coarse-graining. Time series before and after filtering are quite different, indicating that the coarse-graining has a nonnegligible effect on time series.
- For *H* > 0.5, fBm has several low frequency components that are slightly removed by the coarse-graining effect. Time series before and after filtering were fairly similar, indicating that the coarse-graining has a negligible effect on time series.

**Figure 3:**
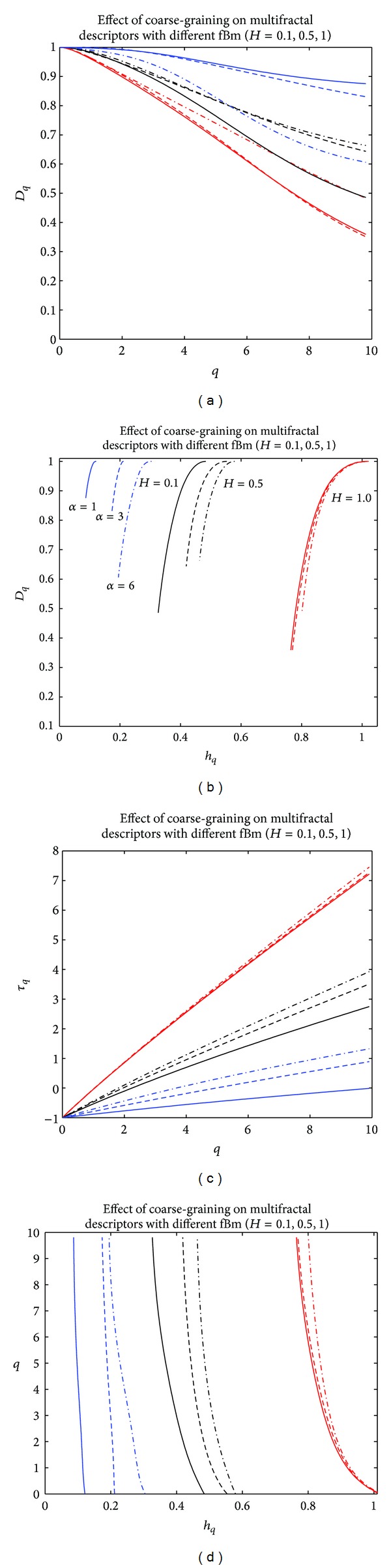
Effects of coarse-graining on multifractal descriptors for different fBm of Hurst exponents *H* = {0.1,0.5,1} with different scale factors *α* = {1,3, 6}. (a) Singularity spectrum *D* versus *q*. (b) Singularity spectrum *D* versus Holder spectrum *h*. (c) Scaling exponent *τ* versus *q*. (d) Holder spectrum.

Figure 3 represents different multifractal descriptors for different fBm of Hurst exponents *H* = {0.1,0.5,1} with different scale factors *α* = {1,3, 6}. The fBm under consideration was composed of 720 samples. The results derived from Figure 3 showed that the anticorrelated fBm of Hurst exponent *H* = 0.1 was more affected by the coarse-graining effect than the correlated fBm of Hurst exponent *H* = 1. These results were compatible with those reported by [26, 27].

### 3.2. Multifractal Analysis

Due to the nonstationary nature of the coarse-grained time series analysed, a short-term procedure was performed. This procedure consisted of evaluating multifractal descriptors from subsignals *y*_*α*_^(*i*)^(*k*) composed of *N* = 720 points (3 min).

Among all the existing methods supplying multifractal descriptors, we used the structure function of order *q*. Although it has been demonstrated theoretically that for certain types of signals the methods based on structure function of order *q* have limitations for *q* < 0, we believe that this type of approach is still worth using because of the following.

- The structure function is by far the simplest method to implement compared to DFA, box counting, and wavelet methods.
- Using *q* < 0 is valuable for analysing very small variations in time series. However, as time series were mostly corrupted by noise, it was impossible to probe small variations in the time series clearly. The practical value of such a negative order *q* was strongly limited by the presence of noise.
- The real signals under consideration were not theoretical signals. This means that mathematical demonstrations operating exclusively on theoretical signals are not systematically applicable in practice.
- Several multifractal analyses showed that it was more possible to discriminate between normal and distressed subjects for *q* > 0 than for *q* < 0. This was particularly the case in (i) [13] where it was shown that the difference between the scaling exponent *τ*(*q*) obtained for healthy and distressed subjects was greater for *q* > 0 than for *q* < 0 and in (ii) [28, 29] where it was clearly shown that for *q* > 0 it was possible to discriminate patients better with atropine than with placebo.

The structure function that we used in this study is defined [23] for *q* > 0 as follows:

(2) $$\begin{array}{c} Q\left(q,\epsilon \right)={\left(\int {\left|y_{\alpha }^{\left(i\right)}(t+\epsilon )-y_{\alpha }^{\left(i\right)}(t)\right|}^{q}dt\right)}^{1/q}. \end{array}$$

This structure function is a length measurement [26] where the term |*y*_*α*_^(*i*)^(*t*+*ϵ*)−*y*_*α*_^(*i*)^(*t*)|^*q*^ reveals a local behaviour while the term (∫⋯*dt*)^1/*q*^ reveals a global behaviour.

If *Q*(*q*, *ϵ*) = *Kϵ* ^*η*(*q*)^, then the scaling exponent *η*(*q*) is expressed (demonstration: log⁡*Q*/log⁡*ϵ* = *η* − (log⁡*K*/log⁡*ϵ*) and lim⁡_*ϵ*→0_(log⁡*Q*/log⁡*ϵ*) = *η*) as

(3) $$\begin{array}{c} \eta \left(q\right)=\underset{\epsilon \to 0}{\lim}\frac{\log\left(Q\left(q,\epsilon \right)\right)}{\log\left(\epsilon \right)}. \end{array}$$

Note that for a fractional Brownian motion of Hurst exponent *H*, the scaling exponent is *η*(*q*) = *H*. From the previous equation, the singularity spectrum *D*(*q*) can be evaluated as follows:

(4) $$\begin{array}{c} D\left(q\right)=q^{2}\frac{d\eta \left(q\right)}{dq}+1. \end{array}$$

The Holder spectrum is written as

(5) $$\begin{array}{c} h\left(q\right)=q\frac{d\eta \left(q\right)}{dq}+\eta \left(q\right). \end{array}$$

Note that this singularity spectrum *D*(*q*) can be obtained through a Legendre transform from *τ*(*q*):

(6) $$\begin{array}{c} D\left(q\right)=q\frac{d\tau \left(q\right)}{dq}-\tau \left(q\right), \end{array}$$

where *τ*(*q*) is another scaling exponent defined by

(7) $$\begin{array}{c} \tau \left(q\right)=q\eta \left(q\right)-1. \end{array}$$

In this case, the Holder spectrum is written as

(8) $$\begin{array}{c} h\left(q\right)=\frac{d\tau \left(q\right)}{dq}. \end{array}$$

**Figure 4:**
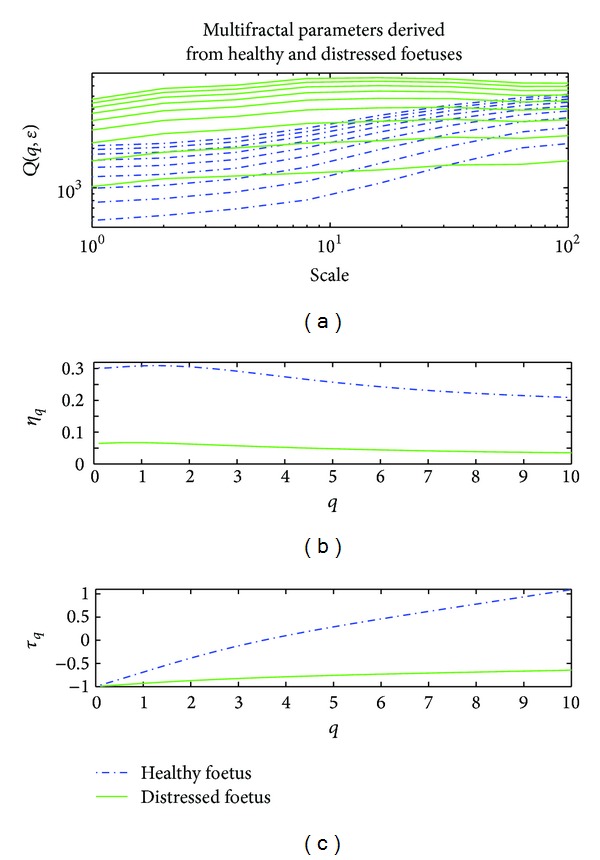
Multifractal parameters for a normal foetus (in blue) and a distressed foetus (in green). (a) Structure function *Q*(*q*, *ϵ*) versus scale. (b) Scaling exponent *η* versus *q*. (c) Scaling exponent *τ* versus *q*. The two scaling exponents were more nonlinear for a healthy foetus than for a distressed foetus.

The structure function *Q*(*q*, *ϵ*) and the scaling exponents *η*(*q*) and *τ*(*q*) for a normal foetus and a distressed foetus are reported in Figure 4 as an illustration.  Figure 4(a) shows that the slopes of the curves obtained for different values of *q* derived from the structure function *Q*(*d*, *ϵ*) were similar for the normal foetus. Similar results were derived for a distressed foetus. Figures 4(b) and 4(c) show that both scaling exponents *η*(*q*) and *τ*(*q*) were more nonlinear for the healthy foetus than for the distressed foetus.

**Figure 5:**
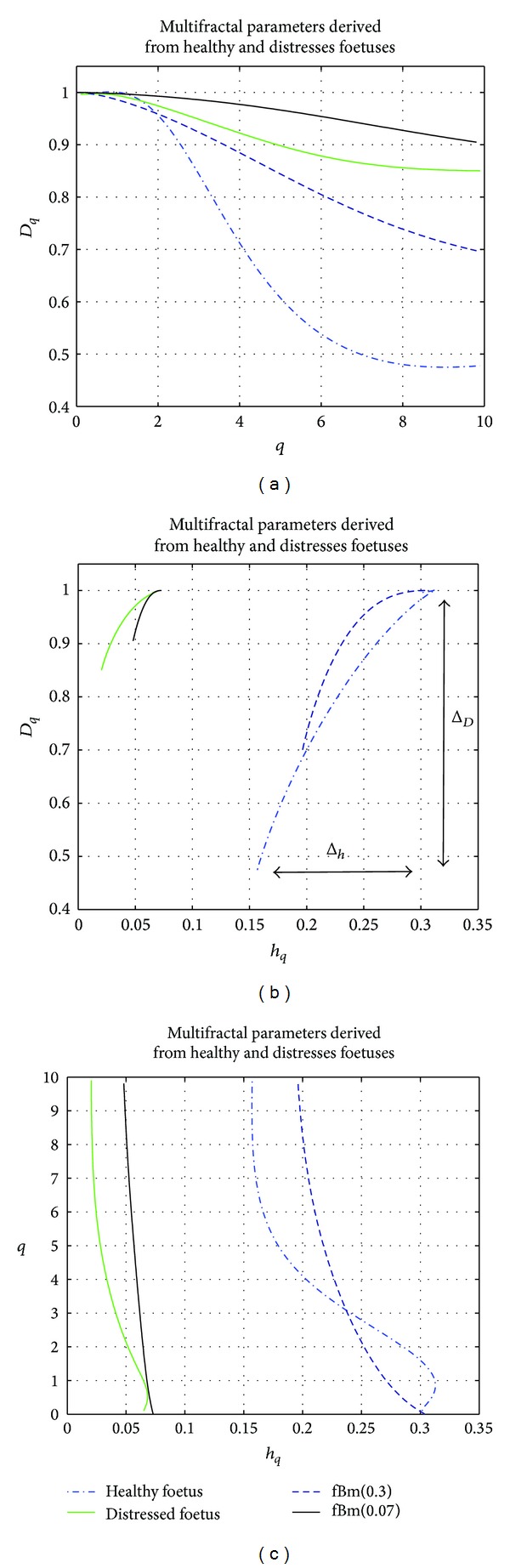
Multifractal parameters for a normal foetus (in blue) and a distressed foetus (in green). (a) Singularity spectrum *D* versus *q*. (b) Singularity spectrum *D*(*q*) versus Holder spectrum *h*(*q*). (c) Holder spectrum *h* versus *q*.

**Figure 6:**
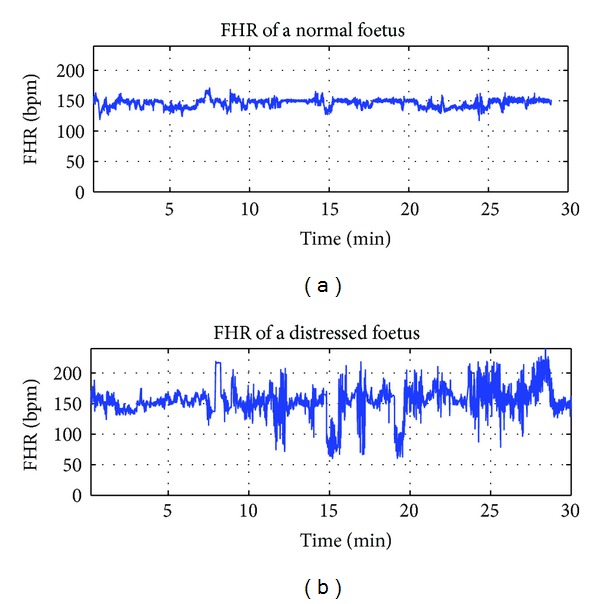
FHR of a normal foetus and a distressed foetus.

Other multifractal descriptors such as the singularity spectrum *D*(*q*) and the Holder spectrum *h*(*q*) are reported in Figure 5. The results set out in Figure 5 were obtained from four different signals: a signal from a distressed foetus of an estimated Hurst exponent *H* = 0.07, a signal from a normal foetus of an estimated Hurst exponent *H* = 0.31, and two fractional Brownian motion (fBm) signals of Hurst exponents *H* = 0.07 and *H* = 0.31. These four signals were each composed of 720 samples. Figure 5 shows that the magnitude of the dynamics of the singularity spectrum *D*(*q*) and the Holder spectrum *h*(*q*) was higher for the healthy foetus than for the distressed foetus. Similarly, the magnitude of the dynamics of *D*(*q*) and *h*(*q*) was higher for foetal signals than for the fBm of the Hurst exponent, as for foetal signals. This corroborates most of the studies based on the analysis of multifractal HRV [12] where a more pronounced multifractal feature for healthy subjects was demonstrated than for distressed subjects. The Holder spectrum for healthy and distressed foetuses decreased with increasing values of *q*, thus supporting the multifractal nature of FHR time series. Such results are consistent with previous similar studies [12, 16]. Note that normal and distressed fetal heart rate time series were reported in Figure 6 as an illustration.

Several measurements were performed in order to quantify the different trends observed in the multifractal indicators *D*(*q*) and *h*(*q*) for different scales *α*.

(i) The relative error RE_1_ (in %) of the Hurst exponent is defined as follows:
  (9) $$\begin{array}{c} \text{R}{\text{E}}_{1}\left(\alpha \right)=\frac{\left|{\overset{-}{H}}_{n}\left(\alpha \right)-{\overset{-}{H}}_{d}\left(\alpha \right)\right|}{{\overset{-}{H}}_{n}\left(\alpha \right)}, \end{array}$$
 where *η*(*q*) = *H* is the Hurst exponent for all *q*. Note that *H* = *η*(1). ${\overset{-}{H}}_{n}$ was the mean Hurst exponent corresponding to the average value obtained for all normal foetuses and ${\overset{-}{H}}_{d}$ was the mean Hurst exponent corresponding to the average value obtained for all distressed foetuses.
(ii) The relative error RE_2_ (in %) of the dynamics of *h*(*q*) is defined as follows:
  (10) $$\begin{array}{c} \text{R}{\text{E}}_{2}=\frac{\left|\overset{-}{\Delta_{hn}}-\overset{-}{\Delta_{hd}}\right|}{\overset{-}{\Delta_{hn}}}, \end{array}$$
 where Δ_*h*_ = max⁡(*h*) − min⁡(*h*) are the dynamics of *h*(*q*), $\overset{-}{\Delta_{hn}}$ being the mean dynamics corresponding to the average value obtained for all normal foetuses and $\overset{-}{\Delta_{hd}}$ the mean dynamics corresponding to the average value obtained for all distressed foetuses.
(iii) The relative error RE_3_ (in %) is defined as follows:
  (11) $$\begin{array}{c} \text{R}{\text{E}}_{3}=\frac{\left|{\overset{-}{D}}_{n}-{\overset{-}{D}}_{d}\right|}{{\overset{-}{D}}_{n}}, \end{array}$$
 where $\overset{-}{D}=\text{mean}(D(q))$ is the mean value of the singularity spectrum, ${\overset{-}{D}}_{n}$ being the mean value corresponding to the average value obtained for all normal foetuses and ${\overset{-}{D}}_{d}$ the mean value corresponding to the average value obtained for all distressed foetuses.
(iv) The relative error RE_4_ (in %) is defined as follows:
  (12) $$\begin{array}{c} \text{R}{\text{E}}_{4}=\frac{\left|\overset{-}{\Delta_{Dn}}-\overset{-}{\Delta_{Dd}}\right|}{\overset{-}{\Delta_{Dn}}}, \end{array}$$
 where Δ_*D*_ = max⁡(*D*(*q*)) − min⁡(*D*(*q*)) is the mean value of the singularity spectrum, $\overset{-}{\Delta_{Dn}}$ being the mean value corresponding to the average value obtained for all normal foetuses and $\overset{-}{\Delta_{Dn}}$ the mean value corresponding to the average value obtained for all distressed foetuses.

## 4. Results and Discussion

From our own dataset composed of one hundred recordings, each time series of 7200 points was coarse-grained for 6 different scales. From each coarse-grained signal, subsignals composed of 720 points and overlapping by 97% were analysed with multifractal tools.

**Figure 7:**
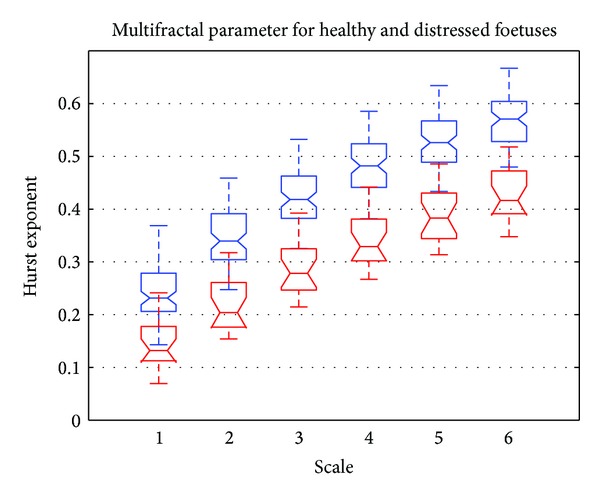
Boxplot of Hurst exponents versus scale. Normal foetus (in blue) and distressed foetus (in red).

Figure 7 shows a boxplot representation of the mean Hurst exponent for different scale values ranging from 1 to 6. Red boxplots correspond to distressed foetuses and blue boxplots correspond to normal foetuses. Figure 7 shows that the mean Hurst exponent for normal foetuses was higher than that obtained for distressed foetuses. This meant that the signatures of distressed foetuses were more irregular and complex than those obtained for normal foetuses. Furthermore, Figure 7 shows that there was sufficient deviation between the mean Hurst exponent to discriminate between normal and distressed foetuses. Figure 7 also shows that the higher the scale, the more regular or filtered the signal (as shown in Figure 1). This corroborated the results of [26], showing that the more filtered the time series the higher the Hurst exponent.

**Figure 8:**
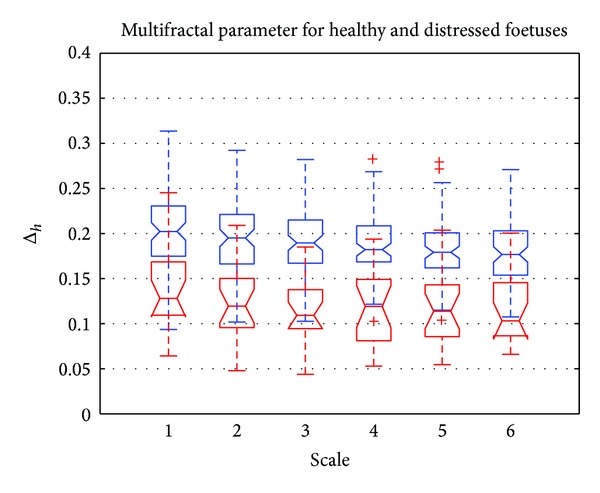
Boxplot of Δ_*h*_ = max⁡(*h*) − min⁡(*h*) versus scale. Normal foetus (in blue) and distressed foetus (in red).

Figure 8 shows a boxplot representation of Δ_*h*_ = max⁡(*h*) − min⁡(*h*). These dynamics are represented for different scale values from 1 to 6. Red boxplots correspond to distressed foetuses and blue boxplots to normal foetuses. Figure 8 shows that the dynamics were higher for normal foetuses than that obtained for distressed foetuses. This meant that the signatures for normal foetuses were more multifractal than those obtained for distressed foetuses. This has already been reported in recent studies such as [12]. Furthermore, Figure 8 shows that there was sufficient deviation between the dynamics to distinguish normal from distressed foetuses.

**Figure 9:**
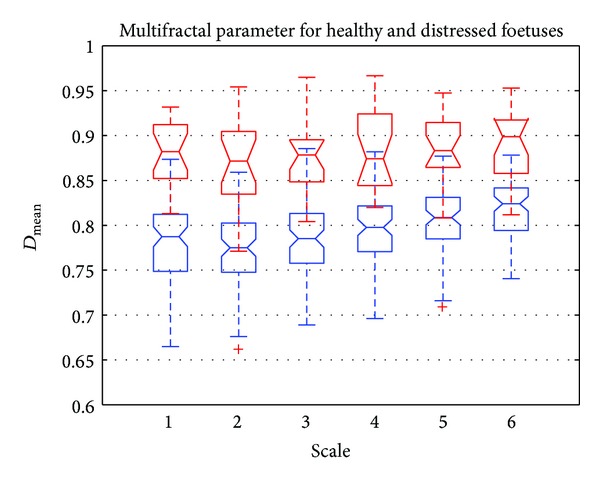
Boxplot of *D*_mean_ versus scale. Normal foetus (in blue) and distressed foetus (in red).

Figure 9 shows a boxplot representation of the mean singularity spectrum $\overset{-}{D}=D_{\text{mean}}$. This parameter was represented for different scale values ranging from 1 to 6. Red boxplots correspond to distressed foetuses and blue boxplots to normal foetuses. Figure 9 shows that $\overset{-}{D}$ was higher for distressed foetuses than for normal foetuses. This meant that the signatures of healthy foetuses were more regular than those corresponding to distressed foetuses. Figure 9 also shows that it was more difficult to discriminate between normal and distressed foetuses. This parameter did not seem to be very relevant. Note also that the higher the scale, the lower the relative error.

**Figure 10:**
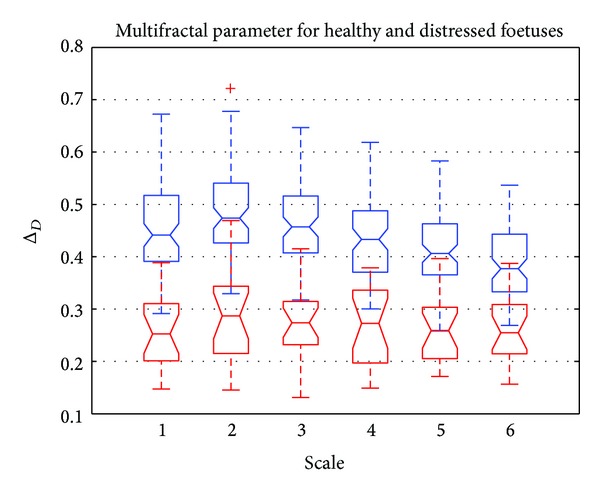
Boxplot of Δ_*D*_ versus scale. Normal foetus (in blue) and distressed foetus (in red).

Figure 10 shows a boxplot representation of Δ_*D*_, that is, the dynamics of *D*. This dynamics is represented for different scale values from 1 to 6. Red boxplots correspond to distressed foetuses and blue boxplots to normal foetuses. Figure 10 shows that Δ_*D*_ was higher for normal foetuses than for distressed foetuses. This meant that the signatures of healthy foetuses were more multifractal than those for distressed foetuses. Figure 10 also shows that there was sufficient deviation between the dynamics to distinguish normal from distressed foetuses. Note also that the higher the scale, the lower the relative error.

To conclude, Table 1 summarizes the relative errors of the four previous parameters. The findings derived from Table 1 showed that the best parameter permitting discrimination between foetuses was RE_4_, followed by RE_1_ and RE_2_. Indeed the best differentiation was obtained for a scale value of 2 for RE_4_ and RE_1_ and a scale value of 3 for RE_2_. This confirms the need to coarse-grain the FHR time series. It is obvious from Table 1 that the higher the scale, the lower the relative error. This requires choosing a maximum scale that is not too high: a value set at 2 seems sufficient whatever the relative error. Furthermore, as the best parameter RE_4_ was sensitive to the multifractal features of the time series analyzed for a scale of 2, this finding confirms the need to analyze FHR from a coarse-grained multifractal point of view. However, note also that as the second discriminative parameter was RE_1_, sensitive to monofractal features set at a scale of 2, then a coarse-grained monofractal approach is also relevant.

Finally, although the present study was quite similar to that presented in [15], our study was different in several ways. First, our study was dedicated to foetuses, whereas [15] was dedicated to adults. Second, our study was based on a much simpler structure function than the other approach that was based on a partition function.

Furthermore, although a large number of research studies have mainly been based on the use of partition functions (DFA, box-counting and wavelet approaches) on the pretext that structure functions do not operate for negative orders, we have shown here (i) that the use of such structure functions is fully justified due to the simplicity of implementation and (ii) that structure functions completely fulfil their role in distinguishing between healthy and distressed foetuses.

Note that, as our proposed methodology was that of investigating offline, we plan to evaluate multifractal descriptors one line in the near future.

## Figures and Tables

**Table 1:** Relative errors of different multifractal parameters between the two groups of foetuses for different scales.

Scale	1	2	3	4	5	6
RE_1_	0.37	0.40	0.33	0.29	0.26	0.24
RE_2_	0.32	0.37	0.38	0.37	0.36	0.35
RE_3_	0.11	0.12	0.11	0.10	0.09	0.08
RE_4_	0.41	0.42	0.41	0.38	0.36	0.33
